# Brain radiotherapy added to first-line immunochemotherapy improves survival in patients with treatment-naïve, driver-negative lung adenocarcinoma and synchronous brain metastases

**DOI:** 10.3389/fonc.2026.1808429

**Published:** 2026-03-26

**Authors:** Rui Wang, Jianxi Zhou, Li Xiao, Hongling Lu, Yaru Kong, Yongchao Yu, Yunchuan Sun, Jiaju Zhang, Yingnan Zhou

**Affiliations:** 1 Department of Head, Neck and Thoracic Oncology, Cangzhou Hospital of Integrated Traditional Chinese and Western Medicine-Hebei, Cangzhou, Hebei, China; 2 Tianjin Medical University Cancer Institute and Hospital, National Clinical Research Center for Cancer, Key Laboratory of Cancer Prevention and Therapy, Tianjin, China; 3 Tianjin’s Clinical Research Center for Cancer, Department of Thoracic Oncology, Tianjin Lung Cancer Center, Tianjin Cancer Institute & Hospital, Tianjin Medical University, Tianjin, China; 4 Department of Hepatobiliary Surgery I, Cangzhou Hospital of Integrated Traditional Chinese and Western Medicine-Hebei, Cangzhou, Hebei, China; 5 Department of Radiotherapy and Chemotherapy, Cangzhou Hospital of Integrated Traditional Chinese and Western Medicine-Hebei, Cangzhou, Hebei, China

**Keywords:** brain metastases, immunotherapy, intracranial progression-free survival, non-small cell lung cancer, overall survival, radiotherapy

## Abstract

**Objective:**

The optimal integration of brain radiotherapy with first-line immunochemotherapy for treatment-naïve, driver-negative lung adenocarcinoma patients with synchronous brain metastases remains unclear. This study assessed the efficacy and safety of this combined strategy.

**Methodology:**

In this retrospective cohort, 172 eligible patients were divided into two groups: the Combination group (immunochemotherapy plus brain radiotherapy, n=86) and the Systemic therapy group (immunochemotherapy alone, n=86). The primary endpoint was overall survival (OS); secondary endpoints included intracranial progression-free survival (iPFS), progression-free survival (PFS), intracranial/extracranial objective response rates (iORR/eORR) and safety.

**Results:**

After a median follow-up of 36.5 months, the Combination group showed significantly longer median OS (23.5 vs. 17.5 months; HR = 0.729, 95%CI:0.538-0.988, P = 0.036) and iPFS (13.6 vs. 7.7 months; HR = 0.699, 95%CI:0.515-0.947, P = 0.017), while unadjusted PFS showed no statistical difference (P = 0.245). iORR was higher in the Combination group (66.3% vs. 46.5%, P = 0.009), with comparable eORR between groups. Multivariate analysis verified brain radiotherapy as an independent favorable factor for OS and iPFS, with consistent benefits across subgroups. Safety profiles were manageable, with no elevated severe immune-related toxicities.

**Conclusion:**

For this patient population, adding brain radiotherapy to first-line immunochemotherapy significantly improves OS and intracranial disease control, without impairing systemic efficacy or increasing severe toxicity, supporting its clinical consideration.

## 1 Introduction

Lung cancer is the leading cause of cancer-related mortality globally, with non-small cell lung cancer (NSCLC) comprising approximately 85% of cases (1). Adenocarcinoma is the most prevalent histological subtype, and a significant proportion of patients present with or develop brain metastases (BM), which are a major determinant of poor quality of life and survival (2, 3). Up to 40% of patients with advanced NSCLC will develop BM, with 10-20% having synchronous metastases at initial diagnosis (4).

The therapeutic landscape for driver gene-negative advanced NSCLC has been transformed by immune checkpoint inhibitors (ICI). First-line treatment combining ICI with platinum-doublet chemotherapy has demonstrated superior overall survival (OS) and progression-free survival (PFS) compared to chemotherapy alone, establishing a new standard of care (5–7). However, patients with active BM were largely underrepresented in these pivotal trials, creating a distinct evidence gap for this common and prognostically adverse subgroup (8, 9).

Local brain-directed radiotherapy, including stereotactic radiosurgery (SRS) and whole-brain radiotherapy (WBRT), remains the cornerstone for controlling symptomatic or sizeable intracranial disease (10). A compelling biological rationale exists for combining radiotherapy with ICI. Radiation can induce immunogenic cell death, release tumor neoantigens, and modify the local tumor microenvironment, potentially converting “immune-cold” brain metastases into “immune-hot” lesions susceptible to systemic immunotherapy—a concept supported by preclinical models and evidence of the abscopal effect (11–13). Retrospective clinical data suggest this combination is feasible and may improve intracranial outcomes, but its definitive impact on OS, particularly when used concurrently in the first-line setting for a molecularly selected cohort, remains inadequately characterized (14–16). Furthermore, the consistency of benefit across clinically relevant subgroups (e.g., defined by PD-L1 expression or BM burden) is unclear.

Therefore, this multicenter retrospective study aimed to evaluate the survival impact, efficacy patterns, and safety of first-line immunochemotherapy combined with brain radiotherapy versus immunochemotherapy alone, in a homogeneous cohort of patients with treatment-naïve, driver-negative lung adenocarcinoma and synchronous BM. We hypothesized that early integration of local radiotherapy would significantly improve OS by enhancing intracranial disease control, without exacerbating systemic toxicity (17, 18).

## 2 Materials and methods

### 2.1 Baseline clinical characteristics

This multicenter, retrospective cohort study was conducted at two tertiary oncology centers in China. We reviewed the medical records of consecutive patients diagnosed with stage IV lung adenocarcinoma between December 2018 and January 2022. Inclusion criteria were: (1) histologically confirmed lung adenocarcinoma; (2) synchronous brain metastases (BM) confirmed by 3D thin-slice contrast-enhanced brain MRI (slice thickness ≤3 mm, covering T1-weighted contrast-enhanced and T2-FLAIR sequences) at initial diagnosis; (3) no targetable driver gene alterations (EGFR, ALK, ROS1, etc.) in tumor tissue; (4) receipt of first-line therapy with a programmed death-1 (PD-1) or programmed death-ligand 1 (PD-L1) inhibitor combined with platinum-based chemotherapy. Key exclusion criteria included: (1) prior systemic therapy for metastatic disease; (2) prior brain surgery or radiotherapy; (3) confirmed leptomeningeal disease; all patients underwent baseline contrast-enhanced brain MRI for preliminary screening, and those with highly suspicious clinical or imaging features received additional whole-spine contrast-enhanced MRI and/or cerebrospinal fluid (CSF) cytology/flow cytometry for definitive diagnosis, with confirmed cases excluded; (4) severe organ dysfunction; (5) active, uncontrolled autoimmune disease requiring systemic immunosuppression (≥10 mg prednisone daily); (6) incomplete clinical or follow-up data. A total of 172 eligible patients were stratified into two groups based on initial treatment strategy: the Combination group (n=86), who received brain radiotherapy plus first-line immunochemotherapy, and the Systemic therapy group (n=86), who received immunochemotherapy alone. Explicit Criteria for Brain Radiotherapy Recommendation: The multidisciplinary tumor board recommended upfront brain radiotherapy based on contemporary clinical guidelines and patient-specific factors to mitigate neurological risks and achieve durable intracranial control: (1) Neurological status: BM-related symptoms (e.g., headache, motor weakness, seizures) or imaging evidence of mass effect. (2) Intracranial tumor burden: Number of BM (SRS/SRT for 1–4 lesions, WBRT for ≥5 or diffuse disease), maximum lesion diameter (>1 cm typically for local therapy), and significant peritumoral edema. (3) Extracranial disease status: Lower extracranial metastasis volume favored aggressive local management. (4) Patient performance status and prognosis: Karnofsky Performance Status (KPS) ≥70 and estimated life expectancy >3 months were required for combined therapy. The final decision integrated these factors with patient preference and technical feasibility.

### 2.2 Treatment protocols

All enrolled patients received uniform, guideline-concordant first-line systemic therapy, with identical regimens, dosing principles, and treatment schedules between the two groups (the only inter-group difference was the administration of brain radiotherapy in the combination group). The systemic regimen consisted of a PD-1 inhibitor combined with platinum-based doublet chemotherapy, administered in 21-day (Q3W) cycles. PD-1 inhibitor dosing: Pembrolizumab 200mg fixed dose, sintilimab 200mg fixed dose, or camrelizumab 200mg fixed dose, administered intravenously on day 1 of each cycle. Agent selection was based on clinical guidelines, drug availability, and patient preference. Chemotherapy dosing: Pemetrexed 500mg/m² body surface area (BSA) intravenously on day 1 of each cycle, plus either carboplatin (area under the curve [AUC] 5) intravenously on day 1, or cisplatin 75mg/m² BSA intravenously on day 1 of each cycle. Induction and maintenance therapy: 4–6 cycles of induction immunochemotherapy were administered, with the exact cycle number determined by treatment response, patient tolerance, and multidisciplinary team consensus. Patients without disease progression or unacceptable toxicity after induction therapy received maintenance therapy with the same PD-1 inhibitor plus pemetrexed (500mg/m² BSA) Q3W, until disease progression, intolerable adverse events, or a maximum treatment duration of 24 months. Dose adjustment: Dose reductions, delays, or discontinuations were performed in accordance with the Common Terminology Criteria for Adverse Events (CTCAE) version 5.0, based on patient hematological/non-hematological toxicity and clinical tolerance. Combination Therapy Group: Patients received brain-directed radiotherapy plus systemic immunochemotherapy, with modality selected by a multidisciplinary team: SRS/SRT: Used for patients with 1–4 brain metastases, delivering high ablative doses per fraction with precise targeting. Common regimens were 15–24 Gy in 1 fraction (smaller lesions) or 27–35 Gy in 3–5 fractions (larger or critically located metastases). WBRT: Used for extensive intracranial disease (≥5 lesions or diffuse involvement), most commonly with 30 Gy in 10 fractions. For select cases with dominant symptomatic lesions, a simultaneous integrated boost (SIB; 40–50 Gy in 10–15 fractions to the gross tumor volume) was added. Radiotherapy timing relative to systemic therapy was concurrent (± 30 days) or sequential (>30 days). For stratified analysis, patients were categorized into two cohorts: SRS/SRT and WBRT-based (conventional WBRT or WBRT+SIB), balancing clinical relevance and statistical feasibility. Radiotherapy Techniques: SRS/SRT was delivered via CyberKnife or Gamma Knife (based on institutional availability and multidisciplinary consensus). WBRT used modern image-guided techniques (primarily VMAT or TOMO), with standard hippocampal avoidance to mitigate neurocognitive risk. Systemic Therapy Group: Patients received immunochemotherapy alone, with local brain therapy reserved for symptomatic progression during follow-up.

### 2.3 Efficacy evaluation

The evaluation of antitumor efficacy was based on the following endpoints and criteria: OS was defined as the time from the initiation of first-line systemic therapy to death from any cause. PFS was defined as the time from treatment initiation to the first documented disease progression at any site (intracranial or extracranial) or death from any cause. iPFS was defined as the time from treatment initiation to the first documented intracranial progression or death attributable to intracranial disease. For the determination of death attributable to intracranial disease in this retrospective analysis, a composite criterion was applied based on comprehensive review of medical records: (a) radiographic evidence of intracranial progression proximate to death in the absence of overwhelming systemic disease, or (b) clinical documentation of neurological deterioration as a primary contributing cause. To address the competing risk of extracranial progression in the iPFS analysis, deaths without clear intracranial attribution and progression events solely at extracranial sites were treated as censoring events at the time of last intracranial disease assessment. Tumor Response was assessed separately for intracranial and extracranial disease. Intracranial response was evaluated using contrast-enhanced brain MRI according to the Response Assessment in Neuro-Oncology for Brain Metastases (RANO-BM) criteria (19). Extracranial response was evaluated using computed tomography (CT) scans according to the Response Evaluation Criteria in Solid Tumors (RECIST) version 1.1 (20).

### 2.4 Safety evaluation

The assessment of treatment-related adverse events (AEs) was conducted as follows: All AEs were identified, documented, and graded according to the Common Terminology Criteria for Adverse Events (CTCAE) version 5.0 (21). The diagnosis and management of immune-related adverse events (irAEs) were guided by established international consensus guidelines (22). Diagnosis of radiation necrosis was based on multidisciplinary review incorporating clinical presentation and multimodal magnetic resonance imaging (MRI) features, in accordance with contemporary diagnostic criteria (e.g., RANO-BM necrosis criteria). Advanced imaging techniques, including perfusion-weighted imaging (PWI) and magnetic resonance spectroscopy (MRS), were utilized when available to aid in distinguishing necrosis from tumor progression.

### 2.5 Follow-up

Following treatment initiation, all patients underwent regular follow-up until the data cutoff date of August 31, 2025. The follow-up protocol included scheduled clinical assessments and radiographic evaluations (contrast-enhanced brain MRI and CT scans of the chest/abdomen) to monitor disease status and treatment response. Radiographic assessments for intracranial disease were typically performed at intervals of 8 to 12 weeks following treatment initiation, or earlier if clinically indicated, in accordance with standard neuro-oncological practice. Disease progression was documented based on these imaging reviews and clinical evaluations. Survival status was verified through hospital records and direct contact, with patients censored at the date of their last confirmed follow-up if no death event was recorded.

### 2.6 Statistical analysis

Statistical analyses were performed using SPSS (version 27.0). Descriptive data were reported as medians (ranges) or frequencies (percentages). Categorical baseline variables were compared using Chi-square or Fisher’s exact tests, and continuous variables using the Mann-Whitney U test. Survival outcomes (OS, PFS, iPFS) were analyzed by the Kaplan–Meier method and log-rank test; hazard ratios (HRs) and 95% confidence intervals (CIs) were calculated via univariate and multivariate Cox proportional hazards models. Treatment effect consistency across prespecified subgroups was assessed using interaction terms in Cox models. A two-sided P < 0.05 was defined as statistically significant. Figures were generated using GraphPad Prism (version 10.0). Propensity score matching (PSM) was performed as a sensitivity analysis to minimize selection bias. Propensity scores were estimated by logistic regression including age, KPS, number and maximum diameter of brain metastases, neurological symptoms, extracranial metastases, and PD-L1 expression. A 1:1 nearest-neighbor matching with a caliper width of 0.2 was applied; covariate balance was confirmed by standardized mean differences (value < 0.10). Survival analyses were replicated in the matched cohort.

## 3 Results

### 3.1 Patient characteristics

During the study period from December 2018 to January 2022, a total of 172 patients were included in the final analysis. The baseline characteristics of the Combination therapy group (n=86) and the Systemic therapy group (n=86) are presented in **Table 1**. No statistically significant differences were observed between the two groups regarding key prognostic factors, including age, KPS, intracranial tumor burden (number and maximum diameter of brain metastases, presence of symptoms), extracranial metastases, or PD-L1 expression (all P > 0.05). The first-line systemic regimens were similarly distributed. In the Combination group, stereotactic radiosurgery was utilized in 60.5% of patients, and 68.6% received radiotherapy concurrently with systemic treatment.

**Table 1:** Baseline characteristics of patients with treatment-naïve, driver-negative lung adenocarcinoma and brain metastases.

Characteristics	Combination group (n=86) n(%)	Systemic therapy group (n=86) n(%)	Statistic	P value
Age			χ²=0.374	0.541
≤60 years	38 (44.2)	42 (48.8)		
>60 years	48 (55.8)	44 (51.2)		
Sex			χ²=0.595	0.440
Male	52 (60.5)	47 (54.7)		
Female	34 (39.5)	39 (45.3)		
Smoking History			χ²=0.441	0.507
Ever	58 (67.4)	62 (72.1)		
Never	28 (32.6)	24 (27.9)		
KPS Score			χ²=0.533	0.466
≥80	78 (90.7)	75 (87.2)		
<80	8 (9.3)	11 (12.8)		
Number of BMs			χ²=1.465	0.481
1	38 (44.2)	42 (48.8)		
2-3	30 (34.9)	32 (37.2)		
≥4	18 (20.9)	12 (14.0)		
Maximum Diameter of BMs			χ²=0.854	0.355
≤2 cm	46 (53.5)	52 (60.5)		
>2 cm	40 (46.5)	34 (39.5)		
Symptoms from BMs			χ²=1.518	0.218
Yes	41 (47.7)	33 (38.4)		
No	45 (52.3)	53 (61.6)		
Extracranial Metastases			χ²=0.732	0.392
Yes	65 (75.6)	60 (69.8)		
No	21 (24.4)	26 (30.2)		
PD-L1 TPS Expression			χ²=0.864	0.649
<1%	29 (33.7)	25 (29.1)		
1-49%	46 (53.5)	52 (60.5)		
≥50%	11 (12.8)	9 (10.5)		
ICI Agent			χ²=1.009	0.604
Pembrolizumab	13 (15.1)	9 (10.5)		
Sintilimab	45 (52.3)	50 (58.1)		
Camrelizumab	28 (32.6)	27 (31.4)		
Platinum-Based Chemotherapy			χ²=1.173	0.279
Pemetrexed + Carboplatin	63 (73.3)	69 (80.2)		
Pemetrexed + Cisplatin	23 (26.7)	17 (19.8)		
Brain RT Characteristics				
RT Modality			N/A	N/A
SRS/SRT	52 (60.5)	-		
WBRT (including boost)	34 (39.5)	-		
RT Timing			N/A	N/A
Concurrent (within ±30 days of ICI)	59 (68.6)	-		
Sequential	27 (31.4)	-		
Prognostic Score				
Lung-molGPA Category			χ²=0.096	0.757
0-2.0 (Poor)	49 (57.0)	51 (59.3)		
2.5-4.0 (Good)	37 (43.0)	35 (40.7)		

BM, brain metastasis; KPS, Karnofsky Performance Status; PD-L1 TPS, programmed death-ligand 1 tumor proportion score; ICI, immune checkpoint inhibitor; SRS, stereotactic radiosurgery; SRT, stereotactic radiotherapy; WBRT, whole-brain radiotherapy; RT, radiotherapy; Lung-molGPA, lung cancer-specific molecular graded prognostic assessment.

### 3.2 Survival outcomes

**Figure 1:**
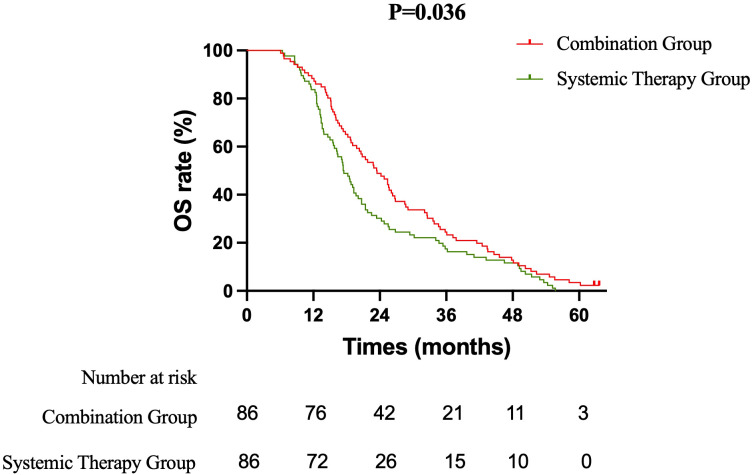
Kaplan-Meier curves for overall survival (OS). The median OS was 23.5 months in the combination therapy group and 17.5 months in the systemic therapy alone group (hazard ratio [HR] = 0.729, 95% CI: 0.538–0.988; log-rank P = .036).

**Figure 2:**
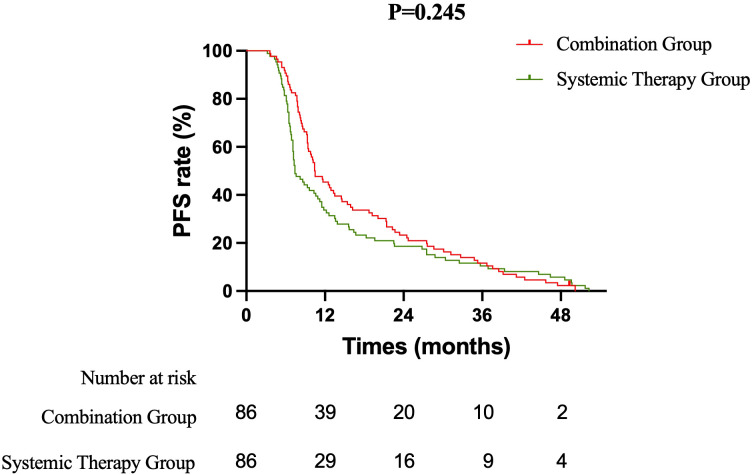
Kaplan-Meier curves for progression-free survival (PFS). The median PFS was 10.5 months in the combination therapy group and 7.4 months in the systemic therapy alone group (HR = 0.840, 95% CI: 0.622–1.135; log-rank P = .245).

**Figure 3:**
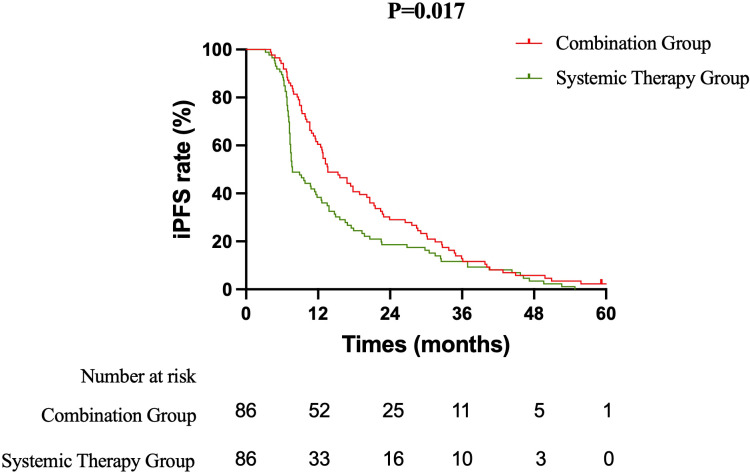
Kaplan-Meier curves for intracranial progression-free survival (iPFS). The median iPFS was 13.6 months in the combination therapy group and 7.7 months in the systemic therapy alone group (HR = 0.699, 95% CI: 0.515–0.947; log-rank P = .017).

The efficacy of the treatment strategies was evaluated based on OS, PFS, and iPFS. OS: After a median follow-up of 36.5 months, the median OS was significantly longer in the Combination therapy group compared to the Systemic therapy group (23.5 months vs. 17.5 months; log-rank P = 0.036). The corresponding 1-year and 2-year OS rates were 88.4% vs. 83.7% and 48.8% vs. 30.2%, respectively. The combination of immunochemotherapy and brain radiotherapy was associated with a 27% reduction in the risk of death (HR = 0.729, 95% CI: 0.538 to 0.988) (**Figure 1**). PFS: The median PFS was 10.5 months in the Combination group versus 7.4 months in the Systemic therapy group (HR = 0.840, 95% CI: 0.622 to 1.135; log-rank P = 0.245). The 1-year PFS rate was 45.3% in the Combination group and 33.7% in the Systemic therapy group (**Figure 2**). iPFS: A significant benefit in intracranial disease control was observed with the addition of radiotherapy. The median iPFS was 13.6 months in the Combination group, nearly double that of the Systemic therapy group (7.7 months; log-rank P = 0.017). The 1-year iPFS rates were 60.5% and 38.4%, respectively. The risk of intracranial progression or death was reduced by 30% with combination therapy (HR = 0.699, 95% CI: 0.515 to 0.947) (**Figure 3**).

### 3.3 Objective response

For intracranial target lesions, the combination therapy group demonstrated superior efficacy. The iORR was significantly higher in the combination group compared to the systemic therapy alone group (66.3% [57/86] vs. 46.5% [40/86]; χ²=6.833, P = 0.009). The iDCR was also significantly improved with the addition of radiotherapy (95.3% [82/86] vs. 83.7% [72/86]; χ²=6.205, P = 0.013) **Table 2**. In contrast, the efficacy against extracranial disease was comparable between the two groups. The eORR was 54.7% (47/86) in the combination group and 51.2% (44/86) in the systemic therapy group (χ²=0.210, P = 0.647). Similarly, the eDCR showed no significant difference (91.9% [79/86] vs. 90.7% [78/86]; χ²=0.073, P = 0.787) **Table 3**.

**Table 2:** Best intracranial response to treatment.

Best intracranial response	Combination therapy group (n=86) n(%)	Systemic therapy group (n=86) n(%)	χ² value	P value
CR	12 (14.0)	5 (5.8)		
PR	45 (52.3)	35 (40.7)		
SD	25 (29.1)	32 (37.2)		
PD	4 (4.7)	14 (16.3)		
iORR	57 (66.3)	40 (46.5)	6.833	0.009
iDCR	82 (95.3)	72 (83.7)	6.205	0.013

CR, complete response; PR, partial response; SD, stable disease; PD, progressive disease; iORR, intracranial objective response rate (CR+PR); iDCR, intracranial disease control rate (CR+PR+SD).

**Table 3:** Best extracranial response to treatment.

Best extracranial response	Combination therapy group (n=86) n(%)	Systemic therapy group (n=86) n(%)	χ² value	P value
CR	8 (9.3)	7 (8.1)		
PR	39 (45.3)	37 (43.0)		
SD	32 (37.2)	34 (39.5)		
PD	7 (8.1)	8 (9.3)		
eORR	47 (54.7)	44 (51.2)	0.210	0.647
eDCR	79 (91.9)	78 (90.7)	0.073	0.787

CR, complete response; PR, partial response; SD, stable disease; PD, progressive disease; eORR, extracranial objective response rate; eDCR, extracranial disease control rate.

### 3.4 Multivariate Cox regression analyses

Multivariable Cox proportional hazards models were constructed to identify independent prognostic factors for OS, PFS, and iPFS, adjusting for key baseline characteristics including age, KPS score, number of brain metastases, presence of neurological symptoms, extracranial metastases, and PD-L1 expression. The results are presented in **Table 4**, **5**, and **6**. After adjustment for these covariates, receipt of combination therapy remained an independent factor associated with significantly improved OS (adjusted HR = 0.682, 95% CI 0.519–0.896; P = 0.006), PFS (adjusted HR = 0.715, 95% CI 0.560–0.913; P = 0.007), and iPFS (adjusted HR = 0.487, 95% CI 0.365–0.649; P<0.001). Other factors independently associated with worse OS included a KPS score <80 (HR = 1.923, P<0.001), the presence of ≥4 brain metastases (HR = 1.705, P = 0.005), and the presence of extracranial metastases (HR = 1.462, P = 0.026). For PFS, independent negative prognostic factors were a KPS score <80 (HR = 1.642, P = 0.002), ≥4 brain metastases (HR = 1.581, P = 0.008), and extracranial metastases (HR = 1.441, P = 0.021). For iPFS, the number of brain metastases was a strong independent prognostic factor, with HRs of 1.520 for 2–3 metastases (P = 0.010) and 2.114 for ≥4 metastases (P<0.001). PD-L1 expression (≥1% vs. <1%) showed a trend towards being a favorable factor for PFS (HR = 0.843, P = 0.096).

**Table 4:** Multivariate Cox regression analysis for overall survival.

Variable	HR	95% CI	P value
Treatment group			
Systemic Therapy Alone	1.000	(Reference)	
Combination Therapy	0.682	0.519 – 0.896	0.006
Age			
≤60 years	1.000	(Reference)	
>60 years	1.158	0.882 – 1.521	0.289
KPS score			
≥80	1.000	(Reference)	
<80	1.923	1.378 – 2.684	<0.001
Number of brain metastases			
1	1.000	(Reference)	
2-3	1.274	0.932 – 1.742	0.129
≥4	1.705	1.173 – 2.478	0.005
Symptoms from brain metastases			
Absent	1.000	(Reference)	
Present	1.245	0.944 – 1.642	0.120
Extracranial metastases			
Absent	1.000	(Reference)	
Present	1.462	1.045 – 2.046	0.026
PD-L1 TPS expression			
<1%	1.000	(Reference)	
≥1%	0.981	0.598 – 1.521	0.121

CI, confidence interval; HR, hazard ratio; KPS, Karnofsky Performance Status; PD-L1 TPS, programmed death-ligand 1 tumor proportion score; RT, radiotherapy.

**Table 5:** Multivariate Cox regression analysis for progression-free survival.

Variable	HR	95% CI	P value
Treatment group			
Systemic Therapy Alone	1.000	(Reference)	
Combination Therapy	0.715	0.560 – 0.913	0.007
Age			
≤60 years	1.000	(Reference)	
>60 years	1.097	0.862 – 1.396	0.453
KPS score			
≥80	1.000	(Reference)	
<80	1.642	1.202 – 2.244	0.002
Number of brain metastases			
1	1.000	(Reference)	
2-3	1.228	0.927 – 1.626	0.152
≥4	1.581	1.124 – 2.224	0.008
Symptoms from brain metastases			
Absent	1.000	(Reference)	
Present	1.185	0.919 – 1.528	0.192
Extracranial metastases			
Absent	1.000	(Reference)	—
Present	1.441	1.058 – 1.962	0.021
PD-L1 TPS expression			
<1%	1.000	(Reference)	
≥1%	0.843	0.583 – 1.447	0.096

CI, confidence interval; HR, hazard ratio; KPS, Karnofsky Performance Status; PD-L1 TPS, programmed death-ligand 1 tumor proportion score; RT, radiotherapy.

**Table 6:** Multivariate Cox regression analysis for intracranial progression-free survival.

Variable	HR	95% CI	P value
Treatment group			
Systemic Therapy Alone	1.000	(Reference)	
Combination Therapy	0.487	0.365 – 0.649	<0.001
Age			
≤60 years	1.000	(Reference)	
>60 years	1.088	0.827 – 1.432	0.550
KPS score			
≥80	1.000	(Reference)	
<80	1.332	0.927 – 1.914	0.120
Number of brain metastases			
1	1.000	(Reference)	
2-3	1.520	1.104 – 2.092	0.010
≥4	2.114	1.458 – 3.065	<0.001
Symptoms from brain metastases			
Absent	1.000	(Reference)	
Present	1.301	0.979 – 1.730	0.071
Extracranial metastases			
Absent	1.000	(Reference)	
Present	1.208	0.854 – 1.708	0.288
PD-L1 TPS expression			
<1%	1.000	(Reference)	
≥1%	1.012	0.827 – 2.077	0.785

CI, confidence interval; HR, hazard ratio; KPS, Karnofsky Performance Status; PD-L1 TPS, programmed death-ligand 1 tumor proportion score; RT, radiotherapy.

### 3.5 Subgroup analysis

**Figure 4:**
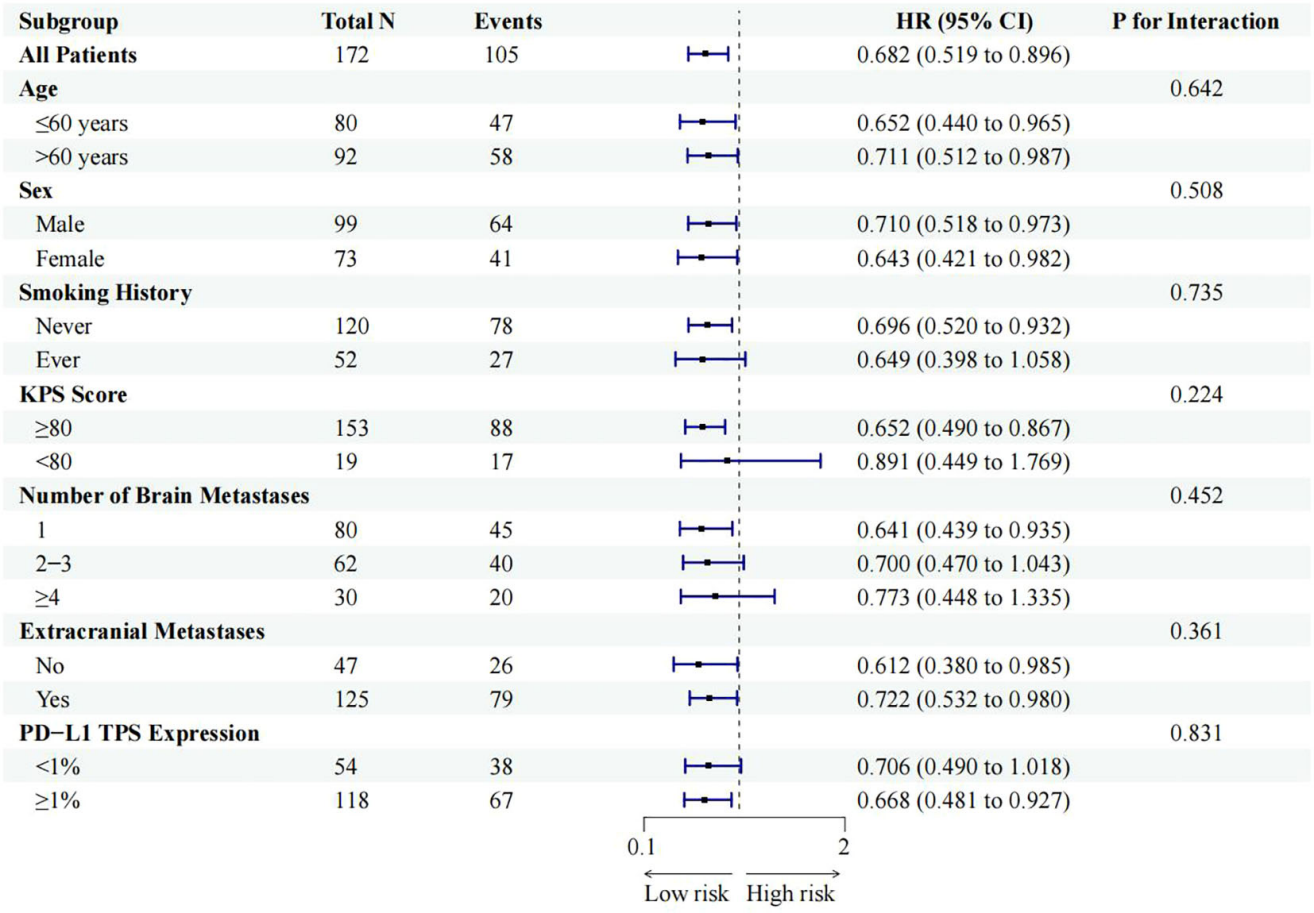
Forest plot of subgroup analyses for overall survival (OS) benefit with combination therapy. Hazard ratios and 95% confidence intervals are shown for each predefined subgroup. P values for interaction are all >.05, indicating no significant heterogeneity in treatment effect across subgroups.

A subgroup analysis was performed to explore the consistency of the OS benefit derived from combination therapy across various patient characteristics (**Figure 4**). The survival advantage favoring the combination therapy group was observed as a consistent trend across all predefined subgroups, including those stratified by age, sex, smoking history, KPS score, burden of intracranial and extracranial disease, and PD-L1 expression level. The P values for interaction were all greater than 0.05, indicating no statistically significant heterogeneity in the treatment effect (all interaction P > 0.05). This analysis suggests that the OS benefit associated with adding brain radiotherapy to first-line immunochemotherapy was consistent regardless of the baseline features examined. Given the clinical relevance of PD-L1 TPS ≥50%, we performed an exploratory analysis in this subset. Among patients with PD-L1 TPS ≥50% (Combination: n=11; Systemic: n=9), the iORR was 81.8% (9/11) versus 55.6% (5/9), and the median iPFS was 18.2 months versus 8.1 months in the combination and systemic therapy groups, respectively. The small sample size in this subgroup precludes formal statistical comparison but suggests a trend towards enhanced benefit with combination therapy among patients with high PD-L1 expression.

### 3.6 Exploratory analysis of radiotherapy modality and timing

To address the heterogeneity in radiotherapy technique, prespecified exploratory analyses were conducted. Compared to Systemic therapy alone, both the SRS/SRT and WBRT-based cohorts demonstrated significantly improved intracranial iPFS, with a consistent trend toward longer OS (**Supplementary Table 1**). Direct comparisons within the Combination group showed no statistically significant differences in OS, iPFS, or progression-free survival between SRS/SRT and WBRT-based modalities, or between concurrent and sequential radiotherapy timing (all P > 0.05; **Supplementary Table 2**). These internal comparisons are acknowledged to be underpowered.

### 3.7 Safety

The safety profile is summarized in **Table 7**. The incidence of grade ≥3 treatment-related AEs was comparable between the combination and systemic therapy groups (27.9% vs. 20.9%, P = 0.555). The spectrum and frequency of immune-related AEs and chemotherapy-related toxicities were similar, consistent with the shared systemic regimen. Expected radiotherapy-specific AEs, including alopecia (32.6%), headache (20.9%), and radiation necrosis (3.5%), were observed only in the combination group. Rates of treatment discontinuation due to AEs were low (7.0% vs. 4.7%, P = 0.515), and no treatment-related deaths occurred.

**Table 7:** Treatment-related adverse events.

Adverse event	Combination group (n=86)		Systemic therapy group (n=86)		Statistic	P value
	All Grades n(%)	Grade ≥3 n(%)	All Grades n(%)	Grade ≥3 n(%)		
Any Treatment-Related AE	78 (90.7)	24 (27.9)	72 (83.7)	18 (20.9)	χ²=0.349	0.555
Immune-related AEs						
Pneumonitis	14 (16.3)	5 (5.8)	10 (11.6)	2 (2.3)	Fisher’s	0.676
Colitis/Diarrhea	9 (10.5)	2 (2.3)	7 (8.1)	1 (1.2)	Fisher’s	1.000
Hepatitis	11 (12.8)	3 (3.5)	9 (10.5)	2 (2.3)	Fisher’s	1.000
Hypothyroidism	17 (19.8)	1 (1.2)	15 (17.4)	0 (0.0)	Fisher’s	1.000
Skin Rash	22 (25.6)	3 (3.5)	19 (22.1)	2 (2.3)	Fisher’s	1.000
Chemotherapy-Related/Systemic AEs						
Hematologic Toxicity¹	45 (52.3)	12 (14.0)	43 (50.0)	11 (12.8)	χ²=0.008	0.929
Fatigue	35 (40.7)	4 (4.7)	29 (33.7)	3 (3.5)	χ²=0.015	0.901
Nausea/Vomiting	15 (17.4)	1 (1.2)	14 (16.3)	2 (2.3)	Fisher’s	1.000
Increased AST/ALT	32 (37.2)	5 (5.8)	28 (32.6)	4 (4.7)	χ²=0.016	0.901
Radiotherapy-specific AEs						
Alopecia²	28 (32.6)	0 (0.0)	-	-	-	-
Radiation Necrosis	3 (3.5)	2 (2.3)	-	-	-	-
Headache³	18 (20.9)	2 (2.3)	-	-	-	-
Treatment Discontinuation due to AE	6 (7.0)		4 (4.7)		χ²=0.425	0.515
Treatment-Related Death	0 (0.0)		0 (0.0)		-	-

AE, adverse event; ALT, alanine aminotransferase; AST, aspartate aminotransferase.

### 3.8 Propensity score-matched analysis

To address potential selection bias, a 1:1 propensity score matching was performed. After matching (70 pairs), baseline characteristics were well-balanced (all standardized differences <0.10; see **Supplementary Table 3**). In this matched cohort, the combination therapy group continued to demonstrate significantly longer median OS (23.9 vs. 17.4 months; HR = 0.710, 95% CI 0.506–0.996; P = 0.041) and iPFS (14.5 vs. 7.6 months; HR = 0.701, 95% CI 0.499–0.984; P = 0.033) compared to the systemic therapy group. The PFS difference remained non-significant (10.6 vs. 7.3 months; HR = 0.819, 95% CI 0.586–1.143; P = 0.228). The corresponding Kaplan-Meier curves are presented in **Supplementary Figure 1**-**3**.

## 4 Discussion

This retrospective study evaluated first-line immunochemotherapy plus brain radiotherapy versus immunochemotherapy alone in treatment-naïve, driver-negative lung adenocarcinoma patients with synchronous brain metastases. The combination significantly prolonged median OS (23.5 vs. 17.5 months; HR = 0.729, P = 0.036) and iPFS (13.6 vs. 7.7 months; HR = 0.699, P = 0.017), and improved iORR (66.3% vs. 46.5%, P = 0.009), with no significant differences in unadjusted PFS, eORR or grade ≥3 treatment-related adverse events between the two groups. The observed survival and intracranial efficacy benefits align with the biological hypothesis of radiotherapy-ICI synergy: radiotherapy disrupts the immunosuppressive microenvironment and blood-brain barrier in brain metastases to facilitate immune cell infiltration and drug delivery, converting “immune-cold” intracranial lesions into “immune-hot” targets for immunotherapy (23, 24). These findings are consistent with prior studies: Chen et al. (25). reported longer OS with concurrent SRS and ICI for brain metastases, and Scoccianti et al. (26). demonstrated superior intracranial local PFS with the combination regimen.

The lack of significant systemic PFS benefit in our study differs from Shaverdian et al. (27)., who observed prolonged PFS and OS with pembrolizumab in patients with prior radiotherapy (KEYNOTE-001 secondary analysis). This discrepancy stems from distinct study populations: our cohort included only treatment-naïve patients with synchronous brain metastases, while their analysis enrolled pre-treated patients with various prior radiotherapy regimens. Consistent with our findings, Singh et al. (28). also found no significant survival benefit with anti-PD-1 therapy plus SRS in NSCLC patients with brain metastases, suggesting systemic efficacy of the combination may be affected by tumor heterogeneity and subsequent lines of therapy.

Regarding safety, the combination regimen did not increase severe adverse events, consistent with reports confirming an acceptable safety profile of intracranial radiotherapy plus ICI, with no elevated risk of radiation necrosis or high-grade irAEs (24, 29). Hubbeling et al. (24). specifically verified no significant increase in radiotherapy-related adverse events in ICI-treated patients versus immunotherapy-naïve controls. Our safety analysis focused on acute toxicities and did not formally assess neurocognitive function (NCF) or patient-reported quality of life (QoL)—key outcomes in brain metastasis management. WBRT is well-established to carry a higher long-term neurocognitive decline risk than SRS (30), and our cohort’s radiotherapy modality selection (based on intracranial disease burden) reflected real-world clinical practice of balancing WBRT’s superior intracranial control for extensive disease against its neurocognitive risks. Hippocampal avoidance is used to mitigate such risks, yet comparative data on NCF and QoL in combined radiotherapy-ICI remain insufficient (31). Future prospective trials should incorporate standardized neurocognitive testing and QoL assessments to clarify the therapeutic ratio of this combination.

First and foremost, the significant improvement in iPFS alongside the non-significant difference in unadjusted composite PFS is biologically expected and fully consistent with the mechanism of action of the study intervention. Brain radiotherapy is a local therapy directed exclusively at intracranial metastatic lesions, with no direct cytotoxic effect on extracranial disease sites. Composite PFS is a combined endpoint that integrates progression events from both intracranial and extracranial compartments. In this study, the first-line systemic immunochemotherapy regimen was completely uniform between the two groups, with no significant differences observed in eORR or eDCR. Extracranial disease progression was therefore a dominant driver of overall composite progression events in both cohorts, meaning that local brain-directed radiotherapy would not be expected to alter the trajectory of extracranial disease or the overall unadjusted composite PFS. This finding does not weaken the study conclusions; rather, it confirms the targeted, compartment-specific efficacy of brain radiotherapy, and reinforces that the observed OS benefit is directly driven by durable intracranial disease control. Beyond this core mechanistic explanation, the dissociation between significant OS benefit and non-significant unadjusted composite PFS is further clarified by additional analyses. Multivariate Cox regression identified combination therapy as an independent favorable factor for improved PFS (adjusted HR = 0.715, P = 0.007) after adjustment for baseline prognostic covariates, indicating that effective intracranial control does confer a favorable effect on overall disease progression risk when baseline imbalances are accounted for. More importantly, robust intracranial control reduces the risk of neurological deterioration and neurological mortality, preserves patient performance status, and enables patients to complete full-course frontline therapy and receive subsequent lines of effective systemic treatment, all of which are established mediators of prolonged OS in this patient population (32). Additionally, radiotherapy-induced immunogenic cell death and synergistic effects with ICI may exert delayed systemic antitumor effects, which have a more substantial impact on long-term survival outcomes than the timing of initial radiographic progression captured by composite PFS (33, 34). PD-L1 TPS is the most well-validated prognostic and predictive biomarker for first-line ICI-based therapy in driver-negative advanced non-squamous NSCLC, and its clinical relevance in the brain metastasis setting has been increasingly validated in recent high-level clinical studies.

From a prognostic perspective, high PD-L1 expression has been consistently identified as an independent favorable factor for survival outcomes in patients with NSCLC brain metastases treated with ICI-based regimens, with intracranial treatment response showing a high degree of concordance with extracranial response in multiple prospective and retrospective cohorts (5, 8, 16). In the present study, PD-L1 TPS ≥1% showed a trend towards improved PFS in multivariate analysis (adjusted HR = 0.843, P = 0.096), which is aligned with the well-established prognostic value of PD-L1 expression in this patient population. The lack of statistical significance for this trend is most likely attributable to the limited sample size of the overall cohort, rather than an absence of biological effect. From a predictive perspective, preclinical and clinical evidence has demonstrated that high PD-L1 expression can augment the synergistic antitumor effect of radiotherapy and ICI. Radiotherapy induces immunogenic cell death, releases tumor neoantigens, upregulates PD-L1 expression in the tumor microenvironment, and enhances effector T-cell infiltration into both irradiated intracranial lesions and systemic disease sites. Tumors with high baseline PD-L1 expression have inherently stronger immunogenicity, and are thus more likely to benefit from the combination of radiotherapy and ICI, with more pronounced intracranial disease control and survival benefits (13, 18). Consistent with this biological rationale, our exploratory subgroup analysis of patients with PD-L1 TPS ≥50% showed a numerically higher intracranial objective response rate (iORR, 81.8% vs 55.6%) and nearly 2-fold longer median intracranial progression-free survival (iPFS, 18.2 months vs 8.1 months) in the combination therapy group compared with the systemic therapy alone group. These findings are also consistent with the results of the landmark C-Brain phase 2 trial, which reported a particularly high intracranial response rate of 85% in patients with PD-L1 TPS ≥50% treated with brain radiotherapy combined with first-line immunochemotherapy (16), and the CTONG 2003 trial, which demonstrated a more significant survival benefit from the addition of radiotherapy to immunochemotherapy in PD-L1-positive subgroups (8). It should be clearly noted that the sample size of the PD-L1 TPS ≥50% subgroup in our study is small (total n=20), which precludes formal statistical comparison and definitive conclusions regarding predictive value. Nevertheless, the consistent trend observed in our cohort, aligned with biological rationale and high-level clinical evidence, supports the hypothesis that high PD-L1 expression may identify a patient subgroup that derives enhanced benefit from upfront brain radiotherapy combined with first-line immunochemotherapy. This highlights the need for large-scale, prospective clinical trials to validate the predictive role of PD-L1 expression in this setting, and to further optimize individualized treatment strategies for patients with NSCLC and synchronous brain metastases.

This study has several inherent limitations associated with its retrospective, non-randomized design, which are systematically acknowledged below. First, the retrospective, non-randomized design carries inherent selection bias. While we applied strict enrollment criteria, multivariate Cox regression, and 1:1 PSM to adjust for known prognostic confounders, unmeasured variables may still have impacted treatment decisions and outcomes. Thus, our findings reflect a robust clinical association rather than definitive causality. Second, heterogeneity exists in radiotherapy modalities (SRS/SRT vs. WBRT) and timing (concurrent vs. sequential). The primary objective of this study was to evaluate the overall clinical value of adding upfront brain radiotherapy to first-line immunochemotherapy, rather than compare differential efficacy between specific radiotherapy strategies, justifying the pooling of radiotherapy subgroups. It is acknowledged that the study is underpowered to detect differences between individual radiotherapy strategies, and all related subgroup analyses are exploratory only. Third, no standardized, formal assessment of NCF and patient-reported QoL was performed, a key limitation given these are core endpoints for brain metastasis treatment. The retrospective design precluded scheduled, validated NCF/QoL assessments across the full cohort, though modern hippocampal-avoidance radiotherapy techniques were used to minimize neurocognitive risk. Fourth, systematic data on post-progression therapies were not fully collected. Subsequent treatments after disease progression are a well-established determinant of overall survival in advanced NSCLC, and incomplete data (primarily from patients receiving post-progression care at external facilities) may introduce confounding. Notably, there was no systematic difference in post-progression treatment accessibility between the two groups, minimizing inter-group bias. Fifth, statistical power is limited by the sample size. No prospective sample size calculation was performed for this retrospective cohort, and the moderate overall sample size, particularly small exploratory subgroups (e.g., PD-L1 TPS ≥50%, radiotherapy modality subgroups), limits definitive conclusions from subgroup analyses, which are exploratory in nature and do not alter the study’s core findings. Sixth, residual confounding cannot be fully eliminated. We adjusted for key variables including the presence of brain metastasis-related neurological symptoms, KPS score, and intracranial tumor burden in multivariate and PSM analyses. However, incompletely quantified factors were not fully accounted for, with potential for residual confounding. Seventh, the generalizability of the findings is limited. This two-center study was conducted in China with an exclusively Chinese patient cohort, and treatment protocols aligned with local clinical guidelines and healthcare settings. Thus, results may not be fully generalizable to other ethnic populations or international healthcare systems, though the core treatment regimens and biological rationale align with global clinical standards. Collectively, these limitations do not negate the core clinical findings of this study, but highlight the need for prospective, randomized controlled trials to validate our results, optimize individualized treatment strategies, and address the identified limitations in future research.

## 5 Conclusion

This retrospective analysis shows that adding brain radiotherapy to first-line immunochemotherapy is associated with prolonged OS and superior intracranial disease control in treatment-naïve, driver-negative lung adenocarcinoma patients with synchronous brain metastases, without a significant increase in severe toxicities. These findings support the consideration of early brain-directed therapy in this patient population. However, given the limitations of this study, particularly the heterogeneity in treatment strategies, the conclusions require validation in prospective randomized controlled trials. Future research should aim to define the optimal radiotherapy modality and sequencing relative to immunotherapy within the combined approach, and to systematically evaluate its comprehensive impact on survival, intracranial control, and neurocognitive function.

## Data Availability

The original contributions presented in the study are included in the article/supplementary material. Further inquiries can be directed to the corresponding author.
