# Supplementary figures and images for: Brain radiotherapy added to first-line immunochemotherapy improves survival in patients with treatment-naïve, driver-negative lung adenocarcinoma and synchronous brain metastases
Source: Front Oncol. 2026 Mar 26;16:1808429. doi: 10.3389/fonc.2026.1808429 (PMC13061658; doi:10.3389/fonc.2026.1808429)

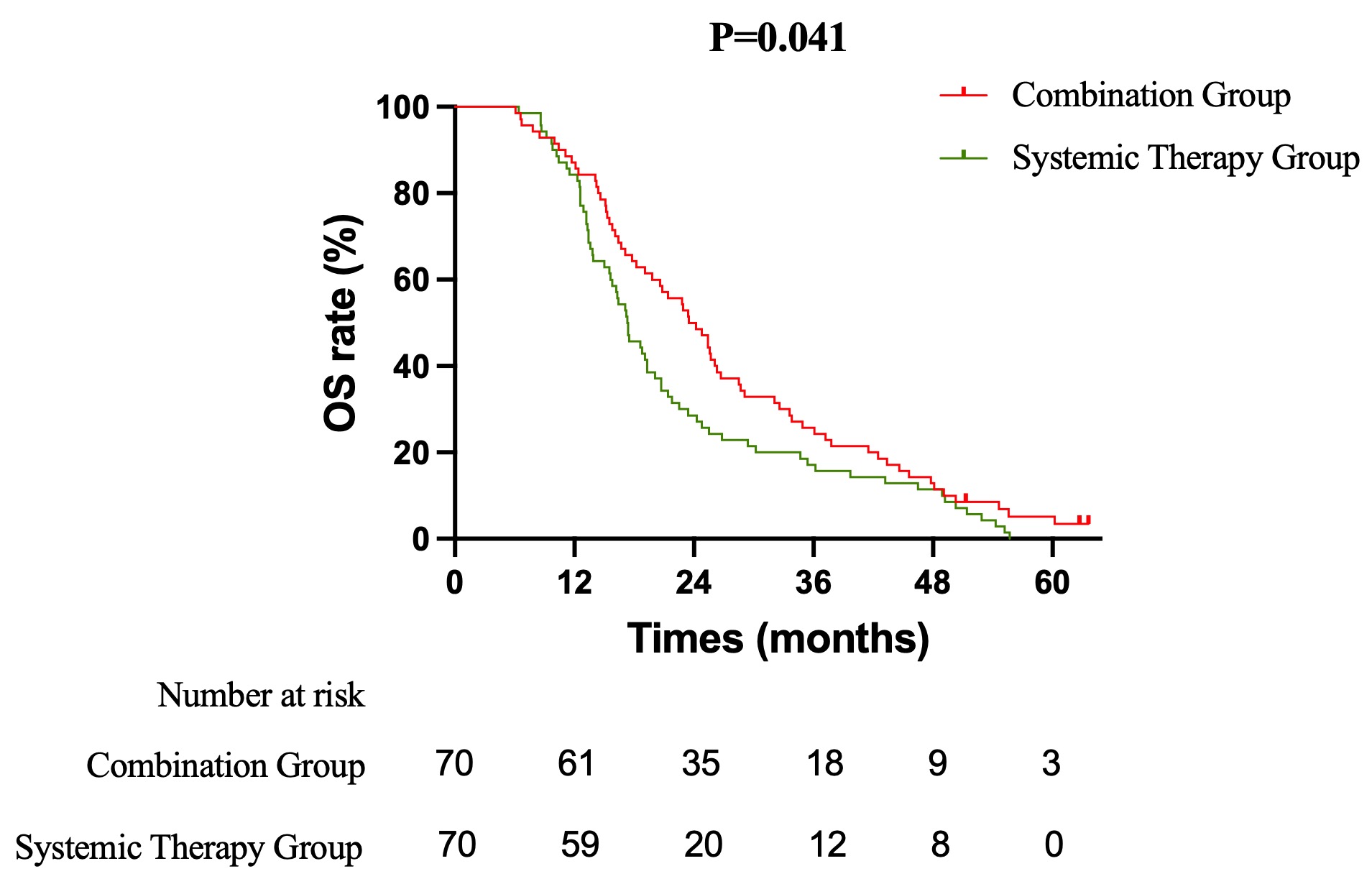

Supplement: Supplementary file 1 [file Image1.jpeg]

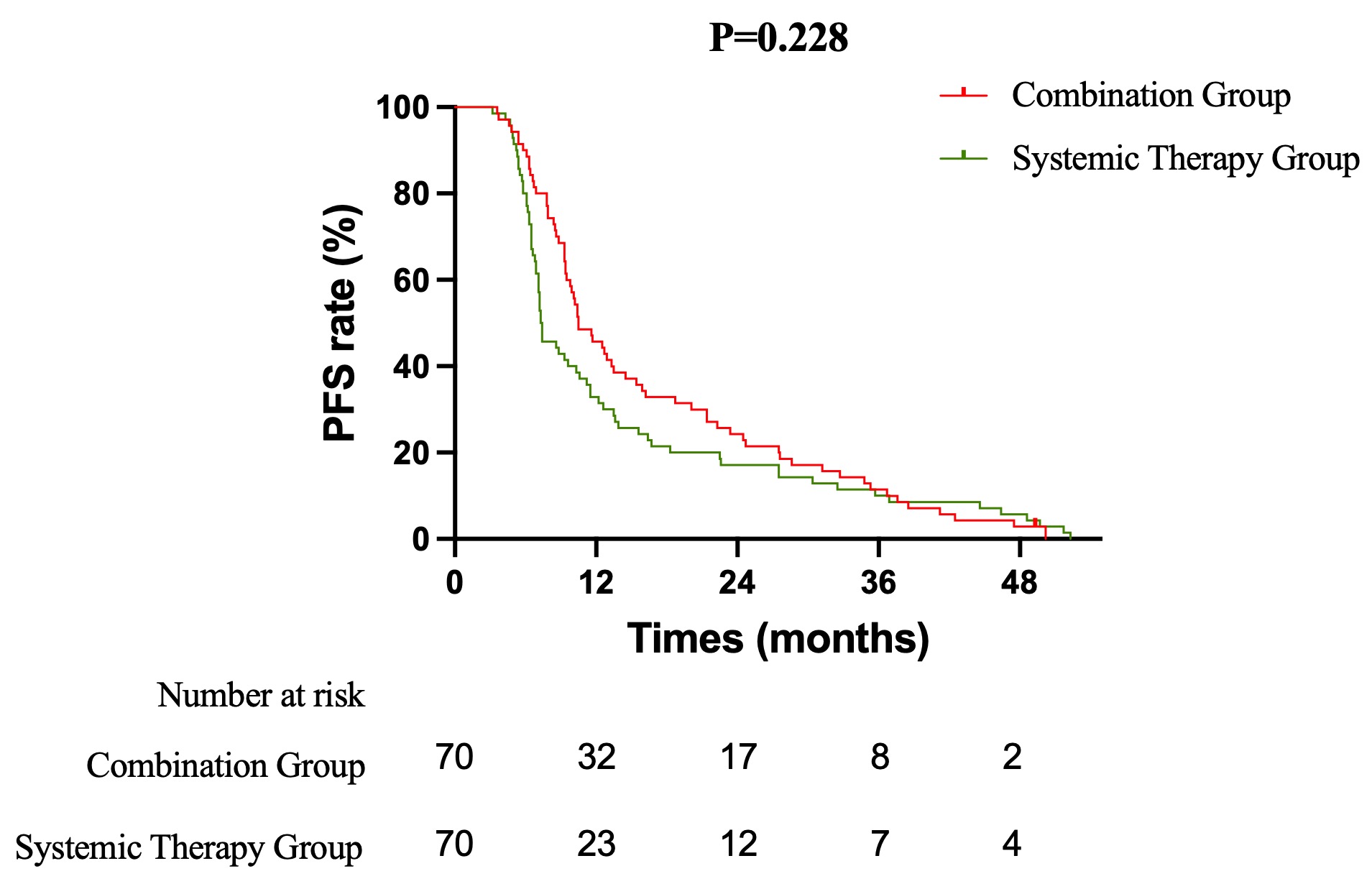

Supplement: Supplementary file 2 [file Image2.jpeg]

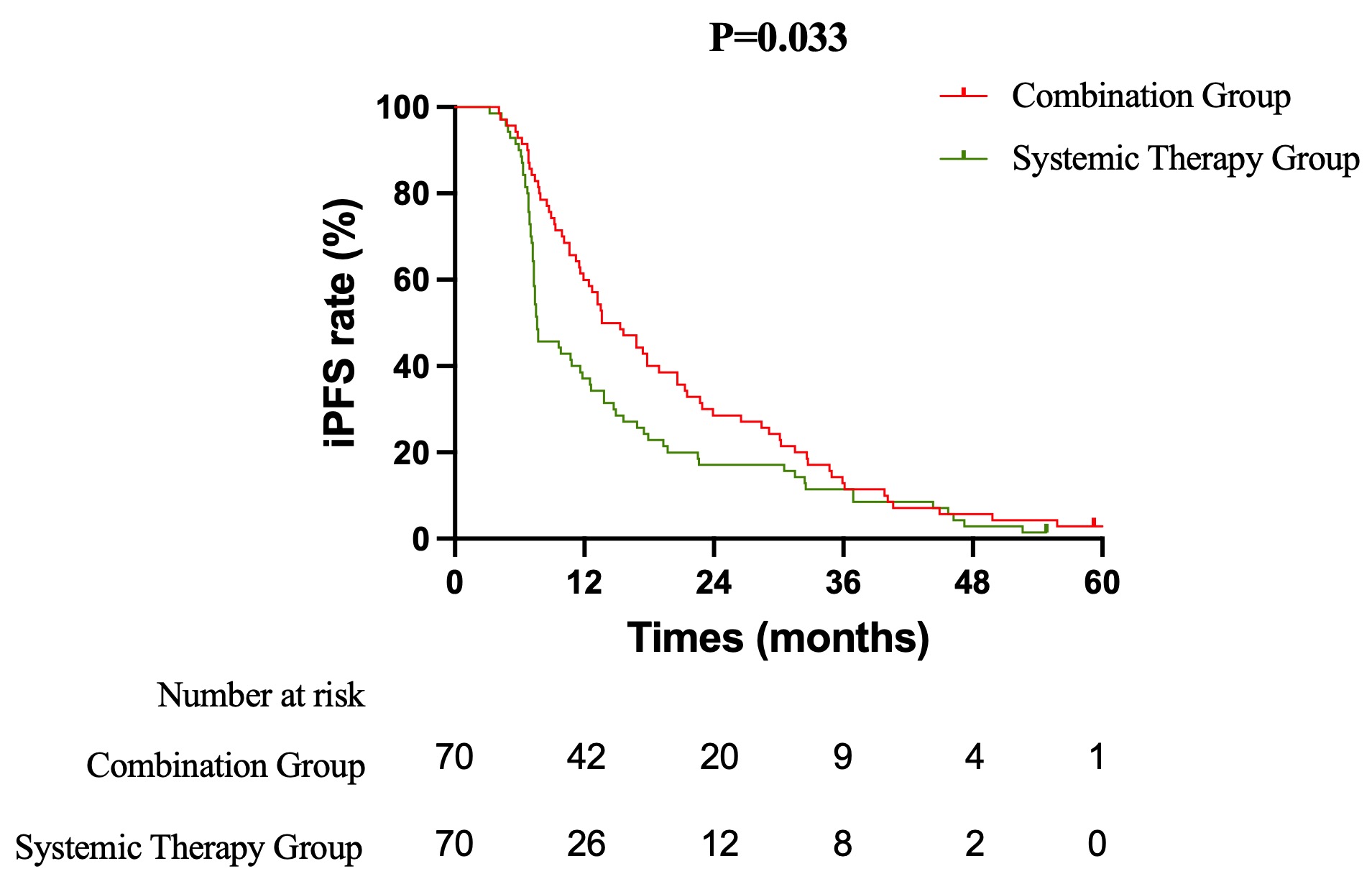

Supplement: Supplementary file 3 [file Image3.jpeg]
