# Supplementary material for: Brain radiotherapy added to first-line immunochemotherapy improves survival in patients with treatment-naïve, driver-negative lung adenocarcinoma and synchronous brain metastases
Source: Front Oncol. 2026 Mar 26;16:1808429. doi: 10.3389/fonc.2026.1808429 (PMC13061658; doi:10.3389/fonc.2026.1808429)
Supplement: Supplementary file 4 [file Table1.docx]

**Supplementary Table 1. Baseline Characteristics After 1:1 Propensity Score Matching.**

| **Characteristics** | **Combination Group (n=70) n(%)** | **Systemic Therapy Group (n=70) n(%)** | **SMD** |
| --- | --- | --- | --- |
| **Age >60 years** | 38 (54.3%) | 36 (51.4%) | 0.058 |
| **KPS <80** | 7 (10.0%) | 8 (11.4%) | -0.045 |
| **Number of BMs** |  |  | **0.032** |
| 1 | 32 (45.7%) | 34 (48.6%) |  |
| 2-3 | 25 (35.7%) | 24 (34.3%) |  |
| ≥4 | 13 (18.6%) | 12 (17.1%) |  |
| **Max Diameter >2 cm** | 30 (42.9%) | 28 (40.0%) | 0.059 |
| **Symptoms from BMs** | 30 (42.9%) | 28 (40.0%) | 0.059 |
| **Extracranial Mets** | 52 (74.3%) | 50 (71.4%) | 0.065 |
| **PD-L1 TPS** |  |  | **0.041** |
| <1% | 22 (31.4%) | 20 (28.6%) |  |
| 1-49% | 38 (54.3%) | 40 (57.1%) |  |
| ≥50% | 10 (14.3%) | 10 (14.3%) |  |
| **Lung-molGPA (Poor)** | 40 (57.1%) | 38 (54.3%) | 0.056 |

PSM, propensity score matching; SMD, standardized mean difference; KPS, Karnofsky Performance Status; BM, brain metastasis; PD-L1 TPS, programmed death-ligand 1 tumor proportion score; Lung-molGPA, lung cancer-specific molecular graded prognostic assessment.
