# Supplementary material for: Brain radiotherapy added to first-line immunochemotherapy improves survival in patients with treatment-naïve, driver-negative lung adenocarcinoma and synchronous brain metastases
Source: Front Oncol. 2026 Mar 26;16:1808429. doi: 10.3389/fonc.2026.1808429 (PMC13061658; doi:10.3389/fonc.2026.1808429)
Supplement: Supplementary file 5 [file Table2.docx]

**Supplementary Table 2. Exploratory Survival Analysis of Radiotherapy Subgroups Compared to Systemic Therapy Alone.**

| **Endpoint and Measure** | **Systemic Therapy Group (n=86)** | **SRS/SRT Subgroup (n=52)** | **WBRT Subgroup (n=34)** | **Concurrent RT Subgroup (n=59)** | **Sequential RT Subgroup (n=27)** |
| --- | --- | --- | --- | --- | --- |
| **OS** |  |  |  |  |  |
| Median, months | 17.5 | 24.5 | 22.0 | 24.8 | 21.5 |
| HR (95% CI) | 1.000 (Reference) | 0.680 (0.470–0.983) | 0.800 (0.535–1.197) | 0.670 (0.470–0.955) | 0.850 (0.560–1.290) |
| P value | — | **0.039** | 0.278 | **0.024** | 0.445 |
| **iPFS** |  |  |  |  |  |
| Median, months | 7.7 | 15.5 | 13.0 | 15.8 | 14.0 |
| HR (95% CI) | 1.000 (Reference) | 0.620 (0.425–0.905) | **0.720 (0.525–0.987)** | 0.600 (0.420–0.857) | **0.740 (0.550–0.995)** |
| P value | — | **0.013** | **0.042** | **0.005** | **0.046** |
| **PFS** |  |  |  |  |  |
| Median, months | 7.4 | 11.0 | 9.5 | 11.2 | 9.0 |
| HR (95% CI) | 1.000 (Reference) | 0.800 (0.560–1.150) | 0.880 (0.590–1.310) | 0.780 (0.550–1.110) | 0.920 (0.610–1.390) |
| P value | — | 0.230 | 0.522 | 0.168 | 0.692 |

SRS, stereotactic radiosurgery; SRT, stereotactic radiotherapy; WBRT, whole-brain radiotherapy; RT, radiotherapy; OS, overall survival; PFS, progression-free survival; iPFS, intracranial progression-free survival; HR, hazard ratio; CI, confidence interval.
