# Supplementary material for: Brain radiotherapy added to first-line immunochemotherapy improves survival in patients with treatment-naïve, driver-negative lung adenocarcinoma and synchronous brain metastases
Source: Front Oncol. 2026 Mar 26;16:1808429. doi: 10.3389/fonc.2026.1808429 (PMC13061658; doi:10.3389/fonc.2026.1808429)
Supplement: Supplementary file 6 [file Table3.docx]

****Supplementary Table 3. Direct Comparisons of Radiotherapy Approaches Within the Combination Therapy Group.****

| **Measure** | **SRS/SRT vs. WBRT** | **Concurrent vs. Sequential RT** |
| --- | --- | --- |
| **Groups (N)** | 52 vs. 34 | 59 vs. 27 |
| **OS** |  |  |
| Median, months | 24.5 vs. 22.5 | 24.8 vs. 22.0 |
| HR (95% CI) | 0.878 (0.534–1.446) | 0.815 (0.472–1.407) |
| P value | 0.624 | 0.462 |
| **iPFS** |  |  |
| Median, months | 15.5 vs. 13.0 | 15.8 vs. 14.0 |
| HR (95% CI) | 0.834 (0.503–1.382) | 0.783 (0.455–1.346) |
| P value | 0.436 | 0.374 |
| **PFS** |  |  |
| Median, months | 11.0 vs. 9.5 | 11.2 vs. 9.0 |
| HR (95% CI) | 0.912 (0.588–1.414) | 0.867 (0.531–1.415) |
| P value | 0.685 | 0.567 |

CI, confidence interval; HR, hazard ratio; iPFS, intracranial progression-free survival; OS, overall survival; PFS, progression-free survival; RT, radiotherapy; SRS, stereotactic radiosurgery; SRT, stereotactic radiotherapy; WBRT, whole-brain radiotherapy.
